# Client and healthcare worker experiences with differentiated HIV treatment models in Eswatini

**DOI:** 10.1371/journal.pone.0269020

**Published:** 2022-05-25

**Authors:** William Reidy, Hervé Nzereka Kambale, Allison B. Hughey, Tengetile Tezzy Nhlengethwa, Janki Tailor, Nomthandazo Lukhele, Simangele Mthethwa, Anita Hettema, Peter Preko, Miriam Rabkin

**Affiliations:** 1 ICAP at Columbia University, New York, New York, United States of America; 2 Department of Epidemiology, Columbia University Mailman School of Public Health, New York, New York, United States of America; 3 Swaziland National AIDS Programme, Ministry of Health, Mbabane, Hhohho, Eswatini; 4 Clinton Health Access Initiative, Mbabane, Hhohho, Eswatini; 5 ICAP at Columbia University, Mbabane, Hhohho, Eswatini; University of the Witwatersrand, SOUTH AFRICA

## Abstract

**Introduction:**

Universal access to antiretroviral therapy (ART) is a cornerstone of Eswatini’s national HIV strategy, and the number of people on ART in the country more than tripled between 2010 and 2019. Building on these achievements, the Ministry of Health (MOH) is scaling up differentiated service delivery, including less-intensive differentiated ART (DART) models for people doing well on treatment. We conducted a mixed-methods study to explore client and health care worker (HCW) perceptions of DART in Eswatini.

**Methods:**

The study included structured site assessments at 39 purposively selected health facilities (HF), key informant interviews with 20 HCW, a provider satisfaction survey with 172 HCW and a client satisfaction survey with 270 adults.

**Results:**

All clients had been on ART for more than a year; 69% were on ART for ≥ 5 years. The most common DART models were Fast-Track (44%), Outreach (26%) and Community ART Groups (20%). HCW and clients appreciated DART, noting that the models often decrease provider workload and client wait time. Clients also reported that DART models helped them to adhere to ART, 96% said they were “very satisfied” with their current model, and 90% said they would recommend their model to others, highlighting convenience, efficiency and cost savings. The majority of HCW (52%) noted that implementation of DART reduced their workload, although some models, such as Outreach, were more labor-intensive. Each model had advantages and disadvantages; for example, clients concerned about stigma and inadvertent disclosure of HIV status were less interested in group models.

**Conclusions:**

Clients in DART models were very satisfied with their care. HCW were also supportive of the new approach to HIV treatment delivery, noting its advantages to HF, HCW and to clients. Given the heterogeneous needs of people living with HIV, no single DART model will suit every client; a diverse portfolio of DART models is likely the best strategy.

## Introduction

**Fig 1:**
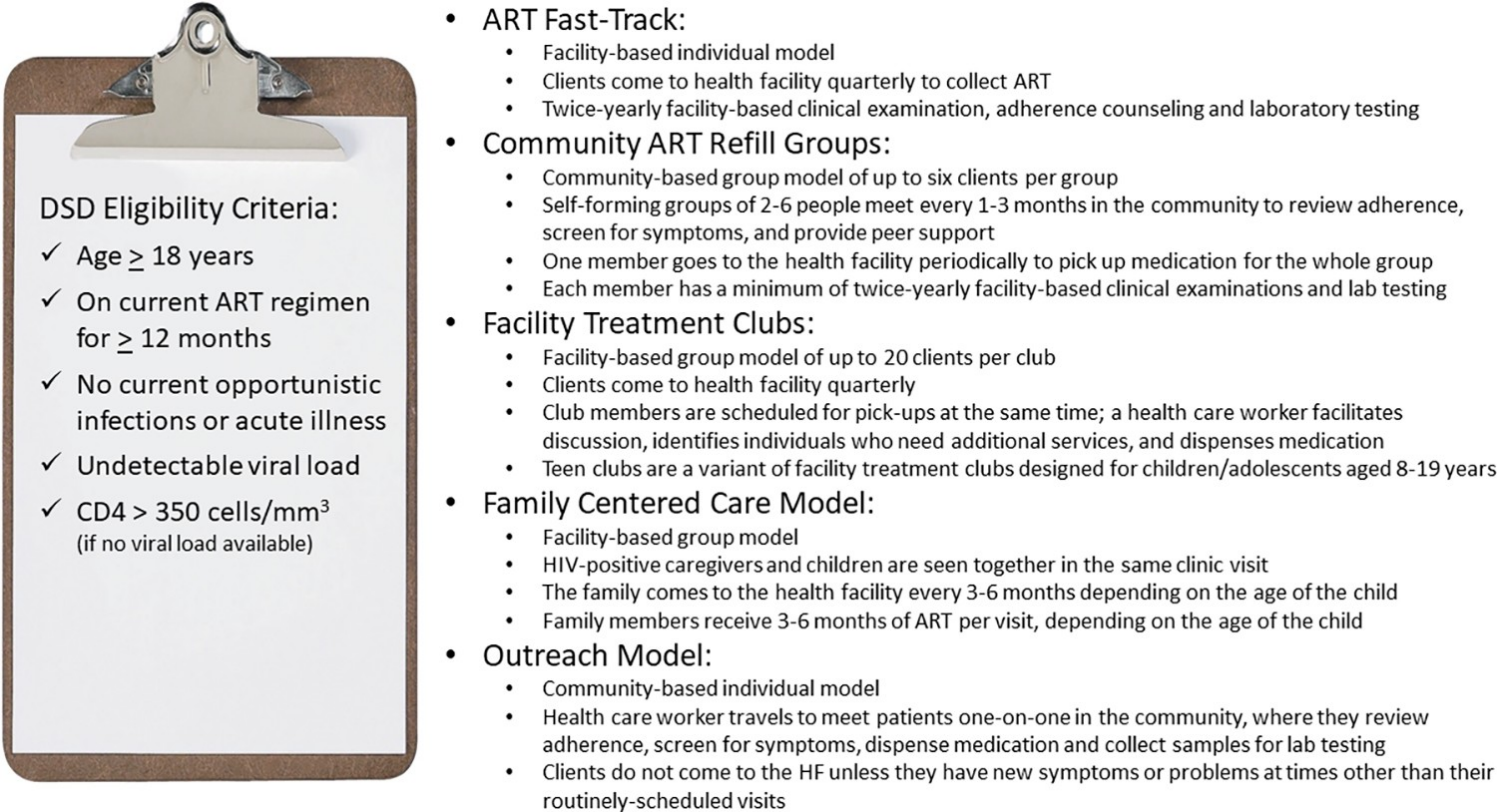
Differentiated ART models and features, and eligibility criteria, Eswatini.

The Kingdom of Eswatini has made remarkable progress scaling up HIV testing, prevention, and treatment services. Although the country has among the world’s highest HIV prevalence, 26.8% among adults in 2021 [1], Eswatini was one of the first countries to achieve the UNAIDS 95-95-95 epidemic control goals [2] and has reduced HIV incidence from 2.4% in 2011 to 0.5% in 2020 [1,3]. Universal access to antiretroviral therapy (ART) is a cornerstone of the national HIV strategy, and the number of people on ART grew from approximately 60,000 in 2010 to over 200,000 in 2020 [4]. To maintain and expand these achievements, the Ministry of Health (MOH) began scaling up differentiated service delivery (DSD) in 2016 [5], including less-intensive differentiated ART models for people doing well on treatment. These models are designed to meet client needs and expectations, and to improve both service quality and efficiency. This evidence-based approach is endorsed by the World Health Organization [6] and by Eswatini’s Integrated HIV Management Guidelines [7] which recommend that adults on ART for at least a year with undetectable viral loads (defined as < 20 copies/ml) and no need for closer monitoring be offered the opportunity to enroll in one of five less-intensive models: Fast-Track, Community ART Refill Groups, Facility Treatment Clubs, Family-Centered Care and Outreach (Fig 1). By September 2021, 89% of people on ART had enrolled in a less-intensive differentiated treatment model [8].

There is an expanding literature documenting the efficiency and impact of differentiated ART [9–15] but less is known about the perspective of clients and healthcare workers (HCW), an important gap in the current evidence base [11,14,16–24]. In response, we designed a mixed-methods study to explore client and HCW perceptions of Eswatini’s differentiated ART models.

## Materials and methods

### Study setting

The study took place between August 2019 and October 2019 at 39 of Eswatini’s 187 health facilities providing ART and their community catchment areas. Health facilities were purposively selected; selection criteria included facility region, health facility type (hospital, health center, clinic), and provision of differentiated ART services.

### Study design

The cross-sectional mixed methods study was designed to explore client and HCW experiences with DSD in general and with specific differentiated ART models, to identify barriers and facilitators of differentiated ART scale-up, and to provide rapid policy-relevant information to MOH (see study protocol in S1 Appendix). Study tools included: (a) a site survey to describe differentiated ART implementation at each health facility; (b) satisfaction surveys with adult clients enrolled in differentiated ART; (c) satisfaction surveys with HCW providing differentiated ART; and (d) key informant interviews with HCW.

### Sampling, data collection and data management

*Site survey*: A senior HCW at each participating health facility was recruited to complete a self-administered paper-based survey capturing relevant facility-level characteristics. The 13-question survey assessed information about staffing, number and volume of clients on ART, available models, and number of clients enrolled in each model and included open-ended questions about future differentiated ART implementation plans and perceived barriers to scale-up. Responses from paper surveys were entered and uploaded by study staff into tablets using a cloud based SurveyCTO (Dobility, Inc., Cambridge, MA, USA) database platform for data management and analysis.

#### Client satisfaction surveys

We enrolled a purposively selected sample of adults aged 18 and older enrolled in differentiated ART at the study sites and presenting for care during the study period, aiming to include at least one client from each model offered by the health facility. Trained study staff administered the client satisfaction questionnaire in siSwati or English and recorded client responses into a tablet computer using SurveyCTO; the questionnaire included 42–60 questions, depending on the differentiated ART model of the participant, and took approximately 20–30 minutes.

#### HCW satisfaction surveys

A purposive sample of at least six HCW from each health facility participated in the HCW satisfaction survey, which included 34 closed-ended questions and 12 open-ended questions. The survey was self-administered in English using paper-based forms and took approximately 20–30 minutes to complete. Data from paper surveys were entered by study staff into a tablet computer using SurveyCTO.

#### HCW key informant interviews

We conducted key informant interviews with 20 purposively selected HCW, all of whom had experience implementing differentiated ART. We intentionally included a variety of HCW cadres to provide perspectives on multiple aspects of differentiated ART within a facility including client consultations, refills and pharmacy operations. Key informant interviews were conducted in siSwati or English using an interview guide and took approximately 20–30 minutes. Interviews were audio recorded and transcribed for analysis.

### Data analysis

Quantitative data from site, client and HCW surveys were exported from Survey CTO, and descriptive statistics including frequencies and percentages were compiled using Stata version 12 (StataCorp LP, College Station, TX, USA). Comparisons of client satisfaction levels across models were done in R [25] using Fisher’s exact test with p-values computed via Monte Carlo simulation (n = 100,000 simulations). Qualitative data from key informant interviews were analyzed using standard thematic analyses, identifying repeated ideas, themes, and thematic narratives through coding and synthesis at increasingly higher levels of abstraction using Dedoose (SocioCultural Research Consultants, LLC, Los Angeles, CA, USA).

### Ethics reviews

The Eswatini National Health Research Review Board (SHR 150/2019) and the Columbia University Medical Center Institutional Review Board (IRB-AAAS6058) approved the protocol. Survey participants provided verbal informed consent and key informant interview participants provided written informed consent.

## Results and discussion

### Facility characteristics

The 39 health facilities were located in each of Eswatini’s four regions: 12 in Hhohho, 8 in Lubombo, 8 in Manzini, and 11 in Shishelweni. Twenty-nine (74%) were clinics, 5 (13%) were hospitals and 5 (13%) were health centers; 28 (72%) were in rural settings (Table 1A). All had provided differentiated ART services since 2016. The Fast-Track model was offered by 35 health facilities (90%). Community ART groups were offered at 22 health facilities (56%), with the Outreach, Family Centered Care and Treatment Club models offered at 12 (31%), 12 (31%) and 9 (23%) health facilities respectively.

**Table 1:** a-d. Characteristics of facilities, survey respondents and key informant interview (KII) participants.

a.) Facilities (n = 39)	n	%	c.) Healthcare worker KII (n = 20)	n	%
Facility type			Cadre		
Clinic	29	*74%*	Nurse	10	*50%*
Hospital	5	*13%*	Nursing Assistant	1	*5%*
Health center	5	*13%*	Medical Officer	1	*5%*
Setting			Expert Client	4	*20%*
Rural	28	*72%*	Pharmacy	3	*15%*
Urban	11	*28%*	Pharmacy assistant	1	*5%*
DART model offered					
Fast-Track	35	*90%*	**d.) Client survey (n = 270)**	**n**	**%**
Community ART Group	22	*56%*	Current DSD model		
Outreach	12	*31%*	Fast-Track	118	*44%*
Family-Centered Care Model	12	*31%*	Outreach	71	*26%*
Treatment Club	9	*23%*	Community ART Group	55	*20%*
			Family-Centered Care Model	17	*6%*
**b.) Healthcare worker survey (n = 172)**	**n**	**%**	Treatment Club	9	*3%*
Cadre			Duration on ART		
Nurse	78	*45%*	1–2 years	32	*12%*
Nursing Assistant	16	*9%*	2–4 years	51	*19%*
Expert Client	53	*31%*	5 or more years	187	*69%*
Counsellor	8	*5%*	Disclosed HIV status to		
Pharmacy	6	*3%*	Family	200	*74%*
Community Health Worker	3	*2%*	Partner	133	*49%*
Medical Officer	2	*1%*	Friend	61	*23%*
Phlebotomist	2	*1%*	No disclosure	12	*4%*
Other	4	*2%*	Travel Time to Facility		
Facility type			0–30 minutes	114	*42%*
Clinic	118	*69%*	31–60 minutes	61	*23%*
Hospital	32	*19%*	>60 minutes	65	*24%*
Health Center	22	*13%*	Employment status		
DSD model training received			Full-time	117	*43%*
Fast-Track	143	*83%*	Part time or casual worker	32	*12%*
Community ART Group	118	*69%*	Unemployed	118	*44%*
Treatment Club	94	*55%*	Prefer not to answer	3	*1%*
Outreach	83	*48%*			* *

### Respondent characteristics

Participants included 20 HCW key informants, 172 HCW survey respondents and 270 client survey respondents. HCW key informant interview and survey respondents included diverse cadres; the majority were nurses (45% and 50% respectively) and expert clients (31% and 20%) (Table 1B and 1C). Client survey participants included 118 (44%) in Fast-Track, 71 (26%) in Outreach, 55 (20%) in Community ART groups, 17 (6%) in Family-Centered Care, and 9 (3%) in Treatment Clubs (Table 1D). Most client respondents (187, 69%) had been receiving ART for 5 or more years and all but 12 (4%) of clients reported having disclosed their HIV status, including 200 (74%) to family and 133 (49%) to a partner.

### DART impact on health facility volume, HCW workload and client time

HCW had diverse perspectives on the impact of differentiated ART models on health facility volume. In the HCW survey, 90 (52%) said volume decreased, 38 (22%) said there was no impact, 25 (15%) said volume increased, and 19 (11%) did not respond.

“What I’ve noted about Fast-Track is it has decreased the number of clients– our patient queues and the waiting time has been reduced a bit as clients on Fast-Track have to only produce their sheets, they don’t have to wait much.”—Senior Nurse, Key informant interview participant

The majority of HCW reported that differentiated ART implementation had an impact on their workload: 110 (64%) said it decreased, 30 (17%) said it increased, 27 (16%) noted no change and 5 (3%) did not respond. Those describing increased workload pointed to specific models, such as Outreach, compared to models requiring less HCW time, such as Fast-Track.

“Before there were no [differentiated ART] models…I’d attend to about 60 clients per day…it has now decreased to about 15 [ART] clients per day. Uh, 15 to 20. So, the workload has decreased drastically, patients are stable. The decrease has been drastic.”—Nurse Assistant, key informant interview participant

“It increases; the outreach increases the workload…How we do it… like yesterday, I was the only present member of my department. When assigning patients, we first have to retrieve files…Record and prescribe…Then we take the prescriptions to pharm techs for them to enter on the computer. And then…then we pack the consignment, on the day before…like yesterday we were busy doing it since after 15:00, you understand?” -Registered Nurse, key informant interview participant

### Client satisfaction

clients reported that advantages of differentiated ART included saving time (187, 69%) and money (54, 20%). In addition, 21 (30%) of the 71 patients in the outreach model cited the lack of need to travel as a benefit, and 20 (28%) of the 72 patients in community ART group and family-centered care models noted the sharing of responsibility for ART pickups as an advantage. The most common dislike reported by clients was the length of wait times (24, 9%). In addition, some HCW highlighted the risk of HIV status disclosure in group models, for example:

“We can’t force clients to be in certain groups. During the morning teaching sessions, [some of] the clients make it clear that they prefer Fast-Track and that they do not want to disclose their statuses….”–Registered Nurse, key informant interview participant

Almost all clients (258, 96%) agreed with the statement “I am very satisfied with my current DSD model;” 266 (99%) agreed that DSD made medication adherence easier; 262 (97%) agreed that DSD made living with HIV easier to manage; and 242 (90%) agreed that they would recommend the model to others (Table 2). Substantial majorities of participants enrolled in each individual model expressed satisfaction within each domain; no significant differences by model were observed.

**Table 2:** Client satisfaction by model.

	Fast- Track		Community ART Group		Outreach		Treatment Club		Family-Centered Care		Total		p-value*
	(n = 118)		(n = 55)		(n = 71)		(n = 9)		(n = 17)		(n = 270)		
	n	*%*	n	*%*	n	*%*	n	*%*	n	*%*	n	*%*	
*"I am very satisfied with my current DSD model"*													
Agree	114	*97%*	53	*96%*	68	*96%*	7	*78%*	16	*94%*	258	*96%*	0.17
Neutral	2	*2%*	2	*4%*	2	*3%*	2	*22%*	1	*6%*	9	*3%*	
Disagree	2	*2%*	0	*0%*	1	*1%*	0	*0%*	0	*0%*	3	*1%*	
*"The DSD model has made it easier to adhere to my ARV regimen"*													
Agree	116	*98%*	55	*100%*	70	*99%*	8	*89%*	17	*100%*	266	*99%*	0.32
Neutral	1	*1%*	0	*0%*	1	*1%*	1	*11%*	0	*0%*	3	*1%*	
Disagree	1	*1%*	0	*0%*	0	*0%*	0	*0%*	0	*0%*	1	*0%*	
*"Being in this DSD model has helped to make it easier to manage living with HIV"*													
Agree	114	*97%*	55	*100%*	68	*96%*	8	*89%*	17	*100%*	262	*97%*	0.51
Neutral	3	*3%*	0	*0%*	2	*3%*	1	*11%*	0	*0%*	6	*2%*	
Disagree	1	*1%*	0	*0%*	1	*1%*	0	*0%*	0	*0%*	2	*1%*	
*"I would recommend this service to others living with HIV"*													
Agree	110	*93%*	50	*91%*	61	*86%*	6	*67%*	15	*88%*	242	*90%*	0.06
Neutral	4	*3%*	3	*5%*	9	*13%*	2	*22%*	1	*6%*	19	*7%*	
Disagree	4	*3%*	2	*4%*	1	*1%*	1	*11%*	1	*6%*	9	*3%*	

*Fisher’s exact test p-value.

## Discussion

Overall, clients were very satisfied with the differentiated ART models they had opted to join and felt that differentiated ART made it easier to adhere to treatment. Overall, 90% of clients said they would recommend differentiated ART to others living with HIV, highlighting convenience, efficiency, and cost savings. These findings are consistent with studies of client preferences for characteristics of differentiated ART models in urban Zimbabwe, where less frequent visits and shorter wait times were prioritized [26]. They are also consistent with studies in Zambia, Malawi and South Africa which found strong client preferences for multi-month dispensing [23,24,27], and with a systematic review of 11 studies of client preferences for HIV service features, which also noted the importance of less-frequent drug pickups and shorter waiting times [28]. Studies of group models, including community- and facility-based adherence clubs in South Africa [29,30] highlight client preferences for convenience, social support and solidarity.

Importantly, many studies find substantial heterogeneity in client preferences, reinforcing the need for diverse differentiated ART models. For example, concerns about inadvertent disclosure in group models make them less attractive to clients concerned about stigma and discrimination, highlighting the importance of stigma reduction training for HCW and thoughtful spatial organization of health facilities to maximize visual and auditory privacy and avoid unintentional disclosure of HIV status [31,32]. Differentiated ART can also be tailored to meet the needs and expectations of specific groups, including key populations [33,34], people with advanced HIV disease [35], pregnant and breastfeeding women [36,37], adolescents and youth [12,38], and migrant/mobile populations [39]. In addition, the importance of kind, person-centered care often outweighs client preferences for convenience and cost-savings [16,26,28].

HCW were also satisfied with differentiated ART implementation, with 64% noting a decreased workload and 52% reporting that health facilities were less congested with shorter wait times following the launch of differentiated ART. This is consistent with surveys of HCW in Malawi [22] and Zambia [24] which found HCW to be supportive of multi-month dispensing; fewer data are available regarding HCW perceptions of other differentiated ART models. As with clients, HCW perceptions varied by differentiated ART model, with Fast-Track decreasing provider workloads and Outreach increasing them.

Limitations of this study include its non-random sampling of facilities and individuals, which reduces the generalizability of findings. Facilities were selected to include a range of facility types and settings across all four regions; however, since this did not involve a randomized process for selection, findings may not be representative of all health facilities or health providers providing differentiated ART in Eswatini. The sampling may have predisposed to clients who were more satisfied with differentiated ART by surveying current differentiated ART clients retained in care, and because clients who were more satisfied with their care may have disproportionately agreed to participate. Key informant interview responses may also be subject to social desirability bias or courtesy bias, as HCW were reporting their perceptions of services provided and endorsed by MOH. Despite these limitations, we believe that the sample size, mixed methods, and inclusion of both HCW and clients resulted in policy-relevant findings and provide a foundation for further implementation science research.

## Conclusions

Clients who chose to be in differentiated ART models were very satisfied with their care. HCW were also supportive of the new approach to HIV treatment delivery, noting its advantages to health facilities, HCW and to clients. Given the heterogeneous needs and preferences of people living with HIV, no single model will work for every client; a diverse portfolio of differentiated ART models for diverse groups is likely the best strategy.

## Supporting information

S1 Appendix Study protocol. (PDF)

S2 Appendix Client survey data. (CSV)

S3 Appendix HCW survey data. (XLSX)

S4 Appendix Site survey data. (XLSX)
