# Supplementary material for: Client and healthcare worker experiences with differentiated HIV treatment models in Eswatini
Source: PLoS One. 2022 May 25;17(5):e0269020. doi: 10.1371/journal.pone.0269020 (PMC9132331; doi:10.1371/journal.pone.0269020)
Supplement: S1 Appendix — (PDF) [file pone.0269020.s001.pdf]

Assessing Client and Healthcare Worker Satisfaction to Inform Implementation and Scale up of Differentiated Service Delivery for People Living with HIV

A Study Protocol Prepared by

Eswatini National AIDS Programme (ENAP)

Contact:

Dr. Herve Nzereka Kambale
Differentiated Service Delivery Coordinator
Eswatini National AIDS Program
hervekambale@gmail.com

Protocol version number: 1.4
Date: 26 June 2019

Investigators and Institutional Affiliations

Institution	Role/s
Eswatini Ministry of Health	• Approval of protocol for submission<br>• Overall stewardship of study and high-level coordination<br>• Participate in review and analysis of data and manuscript development<br>• Approval of all study outputs and materials for dissemination
Clinton Health Access Initiative	• Stakeholder engagement<br>• Study design and protocol development<br>• Execute local IRB submissions and administrative approvals<br>• Hiring, training, and supervision of study staff<br>• Development of study standard operating protocols (SOPs) and data quality assurance strategies<br>• Data collection, data quality assurance, and oversight of study during implementation<br>• Study monitoring and reporting, provide updates to stakeholders and funder reporting<br>• Quantitative data analysis and manuscript development<br>• Dissemination of results within Eswatini<br>• Manuscript development
CQUIN / ICAP	• Technical assistance with development of study protocol and tools<br>• Oversee IRB submission and administrative approvals for Columbia University<br>• Development of study instruments<br>• Qualitative data analysis<br>• Participate in review and analysis of quantitative data and manuscript development
Regional PEPFAR Clinical Partners: EGPAF, URC, ICAP, MSF	• Assist with coordination of data collection within respective regions and health facilities<br>• Provide on-going supervision and support at site level

Investigators and roles

Name	Title/affiliation	Role on study
Dr. Herve Kambale	Differentiated Service Delivery Coordinator, Eswatini Ministry of Health	Principal Investigator:<br>• Protocol development and approval<br>• High-level oversight and monitoring of study implementation<br>• Final approval of all study outputs
Ms. Allison Hughey	Access Program Manager Clinton Health Access Initiative, Eswatini	Co-Investigator:<br>• Overall direction and management of the study<br>• Local IRB submission<br>• Development of protocol, data collection forms, assist with local hiring, financial oversight and donor reporting
Dr. Nomthandazo Lukhele	TB, Hepatitis, HIV, & PMTCT National Program Officer World Health Organization, Eswatini	Co-Investigator<br>• Protocol review and provision of guidance<br>• Study outputs and report review

		• Will not have access to any personal identifying information of participants at any time
Dr. Simangele Mthethwa	National ART Coordinator, Eswatini Ministry of Health	Co-Investigator<br>• Protocol review and provision of guidance<br>• Study outputs and report review<br>• Advise on utilization of study findings to inform program development
Ms. Tengetile Tezzy Nhlengethwa	Access Associate<br>Clinton Health Access Initiative, Eswatini	Co-Investigator:<br>• Coordination of day-to-day study activities<br>• Training and management of data collection team<br>• Data entry, analysis and interpretation<br>• Manuscript and report drafting
Dr. Peter Preko	CQUIN Project Director / ICAP	Co-Investigator:<br>• Review and provide technical input on protocol, study materials and study design<br>• Input on data analysis and manuscript drafting<br>• Will not have access to any personal identifying information of participants at any time
Dr. Bill Reidy	Senior Strategic Information Advisor<br>CQUIN / ICAP New York	Co-Investigator:<br>• Epidemiologist who will provide support for statistical analysis.<br>• Methodological input on design, procedures and forms<br>• Review and provide technical input on protocol, study materials and study design<br>• Columbia University IRB submission<br>• Input on data analysis and manuscript drafting<br>• Will not have access to any personal identifying information of participants at any time
Ms. Andrea Schaaf	Strategic Information Specialist<br>CQUIN / ICAP New York	Co-Investigator:<br>• Review and provide technical input on protocol, study materials and study design<br>• Input on data analysis and manuscript drafting<br>• Will not have access to any personal identifying information of participants at any time
Ms. Siphiwe Shongwe	Clinical Advisor<br>CQUIN / ICAP Eswatini	Co-Investigator:<br>• Review and provide technical input on protocol, study materials and study design<br>• Input on data analysis and manuscript drafting<br>• Will not have access to any personal identifying information of participants during the study

Acronyms

ART	Antiretroviral Therapy
ARV	Antiretroviral
CAG	Community ART Group
DSD	Differentiated Service Delivery
FCCM	Family Centered Care Model
HCW	Healthcare Worker
HIV	Human Immunodeficiency Virus
HTS	HIV Testing Services
IRB	Institutional Review Board
MOH	Ministry of Health
NHRRB	National Health Research Review Board
OI	Opportunistic Infection
PLHIV	People Living with HIV
RHMT	Regional Health Management Team
TB	Tuberculosis

Contents

Investigators and Institutional Affiliations 2
Acronyms 4
Contents 5
Background 7
Justification for the Study 9
General Objectives 9
Specific Objectives 9
Design and Methodology 10
Study Design 10
Client Satisfaction Survey (Appendix 1 and 2) 10
Healthcare Worker Satisfaction Survey (Appendix 3) 10
Healthcare Worker In-depth Interviews (Appendix 4) 11
Senior Healthcare Worker Site Survey (Appendix 5) 11
Study Locations 11
Study Populations 12
Sample Size 12
Recruitment and enrollment 13
Procedures 14
Data Management and Storage 15
Data Analysis 17
Timeline 17
Ethical Considerations 17
Human Subjects 17
Informed Consent 17
Risks and Benefits of Participation 18
Data Ownership 18
Expected Application of the Results 19
Appendices .... **Error! Bookmark not defined.**
Appendix 1. Client Satisfaction Survey (English) .... **Error! Bookmark not defined.**
Appendix 2. Client Satisfaction Survey (siSwati) .... **Error! Bookmark not defined.**
Appendix 3. Healthcare Worker Satisfaction Survey .... **Error! Bookmark not defined.**
Appendix 4. Healthcare Worker In-Depth Interview Guide .... **Error! Bookmark not defined.**
Appendix 5. Senior Healthcare Worker Site Survey .... **Error! Bookmark not defined.**
Appendix 6. Verbal Consent Information Sheet for Clients (English) .... **Error! Bookmark not defined.**

Appendix 7. Verbal Consent Information Sheet for Clients (siSwati) .... **Error! Bookmark not defined.**
Appendix 8. Verbal Consent Information Sheet for Healthcare Workers (English)...... **Error! Bookmark not defined.**
Appendix 9. Verbal Consent Information Sheet for Healthcare Workers (SiSwati) ...... **Error! Bookmark not defined.**
Appendix 10. Written Consent Information Sheet for Healthcare Worker In Depth Interviews (English)............. **Error! Bookmark not defined.**
Appendix 11. Written Consent Information Sheet for Healthcare Worker In Depth Interviews (SiSwati) .... **Error! Bookmark not defined.**
Appendix 12. Study introduction scripts .... **Error! Bookmark not defined.**
Appendix 13. Budget.. **Error! Bookmark not defined.**
Appendix 14. Principal Investigator Curriculum Vitae .... **Error! Bookmark not defined.**

Background

The scale-up of antiretroviral therapy (ART) is one of the world's great public health success stories. The number of people living with HIV (PLHIV) accessing ART in low- and middle-income countries rose from 400,000 in 2003 to 18.2 million in 2016, and an estimated 7.8 million deaths have been prevented by the scale-up of HIV treatment[1]. The Kingdom of Eswatini is close to achieving HIV epidemic control, and has reduced HIV incidence by half by scaling up prevention services and offering ART to all individuals testing positive for HIV, regardless of their immunological and clinical status. As a direct effect, the number of clients on ART has increased from 125,421 in 2014, to 177,156 as of December 2018[2,3]. Continued expansion of the ART programme is required to meet and maintain Ministry of Health (MOH) goals, and it will be critical to ensure efficiency and maintain quality of care as ART coverage continues to rise.

The growing number of clients on ART in Eswatini has however led to overcrowding at health facilities, increasing wait times for clients, overwhelming clinicians, and consequently potentially compromising client satisfaction. The concept of patient satisfaction is an important determinant in the success of any healthcare service "especially in ART units which play a vital role in the lives of thousands of HIV patients[4]". While there is no universally accepted definition of measuring patient satisfaction in the context of health services and service delivery[5], patient satisfaction "is an important goal for both intrinsic patient rights and health outcomes[6]" and is often a combination of expectations and experiences. These include patients' expectations before the visit, during the visit, and extent to which they resolve whatever led them to the health facility[7]. In any situation, a patient reporting on their satisfaction articulates their experience subjectively judging the quality of care[8]. In attempting to measure satisfaction, this is at times done directly through actual experiences of a patient in a clinic or through a study of the patient's attitudes regarding health or care[9].

The importance of measuring satisfaction is also highlighted in the context of Differentiated Service Delivery (DSD). With dwindling resources in global fight to end AIDS, it is noted that "there are many challenges to successfully scaling-up ART and reorienting service delivery toward chronic disease care[10]". Health service researchers have also reported that satisfaction influences patient behavior touching to their likelihood of complying with treatment, following up with appointments and utilizing health services[11]. Satisfaction also adds to patients' retention in care, a critical aspect of any

---

[1] UNAIDS, AIDS by the numbers. Geneva, Switzerland: UNAIDS; November 2016, Retrieved from http://www.unaids.org/sites/default/media_asset/AIDS-by-the-numbers-2016_en.pdf

[2] Eswatini Ministry of Health, 2017 Annual HIV Programme Report

[3] Eswatini Ministry of Health. 2018 Annual HIV Program Report.

[4] Bezuidenhout, Ogunsanwo, and Helberg.

[5] ARM Saifuddin Ekram and Md Fazlur Rahman, 'The Concept "Patient Satisfaction" as It Relates to Contemporary Health CareHealthcare', *TAJ: Journal of Teachers Association*, 19.1 (2016), 5–6 <https://doi.org/10.3329/taj.v19i1.3159>.

[6] Burstein and others.

[7] Ekram and Rahman.

[8] Ashenafi Habtamu, Yibeltal Kifle, and Yohannes Ejigu, 'Client Satisfaction and Its Determinants with Anti-Retroviral Therapy (ART) Services in Public Hospitals of West Wollega Zone , Ethiopia□ : A Cross Sectional Study', 1.March (2017), 1–16.

[9] Afshan and others.

[10] Bezuidenhout, Ogunsanwo, and Helberg.

[11] Habtamu, Kifle, and Ejigu.

success of an ART programme[12]. In Zambia, two challenges faced by their health system that have been linked to reported low patient satisfaction include challenges in health-seeking behavior and adherence to treatment, particularly for HIV[13].

In a study conducted at Gert Sibande Hospital in Mpumalanga, South Africa, which hosts the largest ART Programme in the world, they found that some of the factors which contributed to satisfaction included "the availability of medicines, knowledge regarding how to take medication and general satisfaction with providers" while contributors to dissatisfaction included "waiting too long, confidentiality issues, shortage of staff and dirty toilets"[14]. Oftentimes, indicators of dissatisfaction are interconnected - in cases where clients complain about long wait times, this is related to human resource shortages of doctors and nurses[15]. At times, shortages are experienced because at some ART sites there are no specific doctors purely dedicated to ART[16]. As a result, most of the clients are seen by the same healthcare provider each time they visit the facility. In contrast, a study in Mexico, found that a that there was a relationship between the physician/patient relationship and adherence to ART to which those who were dissatisfied with that relationship were more likely to miss some doses of ART[17]". It remains the case that any improvements in patient satisfaction through service improvements must be cognizant of the continuous challenges posed by the health sector's competing priorities and diminishing resources[18].

Gaps in program quality, such as suboptimal retention rates, threaten both individual client outcomes and public health goals[19]. In response to these challenges, new global guidelines support the use of DSD, moving away from a "one-size-fits-all" facility-based model towards different algorithms and programmatic designs for diverse groups of HIV-positive individuals while maintaining the principles of the public health approach[20,21]. DSD is hypothesized to increase programmatic efficiencies while also improving client and healthcare worker (HCW) satisfaction.

Following a successful pilot of DSD conducted between 2014 and 2015, the Eswatini MOH has adopted innovative strategies to differentiate services for diverse clients. In 2016, the country developed specific DSD guidelines and standard operating procedures to guide DSD implementation. In addition, DSD was included in the 2018 Integrated HIV Management Guidelines, which recommend that stable clients who have been on ART for at least a year and have

---

12 . Sunita and others, 'A Cross Sectional Study to Assess the Satisfaction Level among People Living with HIV/AIDS in a Hilly State of Northern India', *International Journal Of Community Medicine And Public Health*, 5.6 (2018), 2373 <https://doi.org/10.18203/2394-6040.ijcmph20182161>.

13 Burstein and others.

14 Bezuidenhout, Ogunsanwo, and Helberg.

15 Bezuidenhout, Ogunsanwo, and Helberg.

16 Selente Bezuidenhout, Damilola A. Ogunsanwo, and Elvera A. Helberg, 'Patient Satisfaction at Accredited Antiretroviral Treatment Sites in the Gert Sibande District', *African Journal of Primary Healthcare and Family Medicine*, 6.1 (2014), 1–6 <https://doi.org/10.4102/phcfm.v6i1.627>.

17 Diana Pérez-Salgado and others, 'Satisfaction with Healthcare Services and Adherence to Antiretroviral Therapy among Patients with HIV Attending Two Public Institutions', *Revista de Investigacion Clinica; Organo Del Hospital de Enfermedades de La Nutricion*, 67.2 (2015), 80–88.

18 Bezuidenhout, Ogunsanwo, and Helberg.

19 El-Sadr WM, Barker P, Rabkin M, Pillay Y, Birx D. Putting quality at the heart of HIV programs, AIDS 2015;29 (Suppl 2):S119-S120

20 Duncombe C, Rosenblum S, Hellmann N, Holmes C, Wilkinson L, Biot M et al. Reframing HIV care: putting people at the centre of antiretroviral delivery. Trop Med Int Health. 2015;20(4):430-47

21 El-Sadr WM, Rabkin M, De Clock KM. Population health and individualized care in the global AIDS response: Synergy of conflict ? AIDS 2016;30:2145-2148. PMID: 27367489. Doi: 10.1097/QAD.0000000000001192

undetectable viral load should be offered an opportunity to choose among available DSD models as a standard package of care for adults and adolescents[22].

The DSD models currently offered in Eswatini include:

- **ART Fast Track:** clients receive ART refills four times per year but only receive clinical reviews, laboratory testing and adherence counselling twice per year
- **Community ART Groups (CAGs):** self-formed groups of 2-6 clients take turns visiting the facility to get refills on behalf of the other group members, reducing the number of annual visits from four to two. Unless unwell, every member must have at least one clinical review in six months.[23]
- **Facility Treatment Clubs:** groups of clients meet at the facility for group counselling, support and ART collection; may also include income generating activities
- **Teen Clubs:** groups of children and adolescents, age 8-19 years, who meet at the facility for group counselling, psychosocial support, and if stable, ART collection
- **Outreach models:** mobile clinical teams from facilities take the available services directly to the community
- **Family Centered Care Model (FCCM):** caregivers and their children living with HIV are seen together in one clinic visit to promote ART initiation and retention in families and provide more efficient care - this model of care is being piloted in four sites currently

While these innovative strategies are being implemented at different levels of the health system in Eswatini, they have not been systematically evaluated to assess whether or not they lead to improved client and HCW satisfaction. Lessons learned from this study will inform strategies and activities to be adopted for scaling up of DSD in the Kingdom of Eswatini.

Justification for the Study

The Eswatini MOH and implementing partners will conduct this study to explore client satisfaction amongst individuals enrolled in DSD models and provider satisfaction amongst HCW at facilities providing DSD services. The results of this study will also allow the MOH to better understand how DSD models have influenced perceived HIV service quality (on the part of clients) and workload (on the part of HCW). Insights from the study will inform MOH scale-up of DSD in line with the Eswatini National Health Research Agenda to research ways to improve existing interventions for retention and maintaining viral suppression.

General Objectives

The purpose of the study is to assess client and HCW satisfaction with the new DSD models being implemented in Eswatini and to identify facilitators and barriers to successful DSD scale-up from the perspective of these populations.

Specific Objectives

The specific objectives of this study are:

1. To assess HCW satisfaction with the implementation of DSD in Eswatini
2. To explore HCW perceptions regarding barriers and facilitators of DSD implementation
3. To assess client satisfaction with the implementation of the five different DSD models
4. To identify client's perspectives of the barriers to DSD Scale up

---

[22] Swaziland Integrated HIV Management Guidelines, 2018

[23] Standard Operating Procedures for Implementing Community-centered Models of ART Service Delivery (CommART) in Eswatini, 2016

5. To understand client satisfaction and needs under each DSD model to inform a differentiated service delivery model that will better promote retention in care among PLHIV.

Design and Methodology

Study Design

This is a cross-sectional, mixed methods study.

The study will include satisfaction surveys with clients enrolled in DSD, satisfaction surveys with HCW providing DSD, and key informant interviews with HCW providing DSD. In addition, study staff will conduct structured site surveys with senior HCWs to describe the ART services, number of clients on ART, DSD models offered and number of clients enrolled under each model at their health facilities.

Client Satisfaction Survey (Appendix 1 and 2)

A purposive sample of clients enrolled in DSD at study sites will be invited to complete a client satisfaction survey. The survey will be administered by study staff using an electronic form on a password protected tablet. All study staff will be bilingual in English and the local language, siSwati. Clients will be given the option to choose which language they prefer to use when participating in the survey.

Survey questions will include domains on:

- Basic sociodemographic data including age, sex, level of education
- Current HIV care and treatment services, including ART regimen, length of time on ART, length of time in DSD and current DSD model
- Self-reported adherence to medication and retention/missed appointments
- Satisfaction with current HIV services, including wait time, convenience, HCW knowledge and skills, HCW attitudes and courtesy, and DSD model
- Comparison of satisfaction in current DSD model with previous mainstream model of care
- Perceived barriers and facilitators to DSD participation

The survey will not collect participant name, date of birth, clinic idenitification number, or any other personally-identifying information, and the surveys will not be linked to any client's clinical data. Participants will have questions read to them in SiSwati or English in a quiet private area of the health care facility or community. Participants will respond verbally to survey questions in SiSwati or English and have the responses recorded by a study data collector directly into the tablet.

Healthcare Worker Satisfaction Survey (Appendix 3)

All healthcare workers providing ART services at DSD study sites will be invited to complete a HCW satisfaction survey. The survey will be self-administered by HCWs on paper-based forms. No names, or other personally-identifying information, will be collected in this survey.

Survey questions will include domains on:

- Basic socioeconomic demographic data, including age and sex
- Professional information: HCW cadre, length of time working at health facility, length of time providing DSD, type of DSD training received
- Satisfaction with work environment, including supervision, support, resources, workload
- Comparison of current work environment with that prior to implementation of DSD
- Ease of adherence to DSD guidelines, including eligibility criteria

- Perceived barriers and facilitators to DSD implementation

Healthcare Worker In-depth Interviews (Appendix 4)

In addition to the structured satisfaction survey, HCWs will be purposively sampled based on experience with DSD in their facility and asked to participate in in-depth interviews by trained qualitative data collector A semi-structured in-depth interview with each HCW will be conducted using an interview guide included in appendix 4. These interviews will elicit HCW perspectives on the application of and ability to adhere to the DSD eligibility criteria for their clients. Interviews will be audio recorded and transcribed for analysis. No respondent names or other personally-identifying information will be collected during the interview.

Senior Healthcare Worker Site Survey (Appendix 5)

A Senior HCW will be purposively selected requested to complete a Site Survey based on their knowledge and level of oversite of the facility. The individual must have some seniority so as to answer the questions that require them to know some of the following information: average number of clients attended to at the facility; number of clients on ART; number of clients in the various DSD models; and average wait time at the facility (appendix 5). The HCW will complete a self-administered, paper-based survey at a location of their choice. Respondent names will not be collected on the survey, though the name of the health facility will be collected. However, no potentially sensitive information, including personal opinions, will be collected on this survey.

Study Locations

The study will be conducted at 39 purposively selected health facilities and their respective community catchment areas to include CAG meetings and outreach sites, where applicable. Final approval for participation by the health facility will be given by the MOH Regional Health Management Team (RHMT). Health facilities were purposively selected based on the aim of the client survey, which is to inform the future development of differentiated care models in Eswatini and similar settings for PLHIV. The following steps were taken to determine which sites should be included in the study:

1. Gathered and re-collated all data from the DSD Baseline Assessment conducted by the MOH in 2017 and 2018.
2. Organized all information according to the four geographic regions: Manzini, Hhohho, Shiselweni and Lubombo.
3. Selected the 20 existing DSD Quality Improvement Project sites under each of the geographic regions, purposefully selecting their information for review. All these sites were automatically prioritized for their respective regions.
4. Created a list of the top five facilities from the master facilities list sorted according to region and the number of DSD models offered - prioritizing facilities that offer 3 or more DSD models.
5. From the brief list of 5 to 10 facilities ultimately selected per region, ensured that all fully represent;
    a. Hospital
    b. Health center
    c. High volume Clinic
    d. Low volume clinic
    - Selections for each site were based on the facility offering the model needed
    - In the instance that no facility within the top 5 per region was offering a particular model, the master facility list was reviewed and an additional facility was selected to represent that model type
    - For cases where the data remained unavailable because the model was not offered by the appropriate facility guidance was sought from implementing partners on which facilities are now offering the model of care after the initial baseline assessment

The following sites in table 1 below have been proposed for inclusion as study sites based on the selection process described above:

Table 1. Proposed study sites

Hhohho Region	Lubombo Region	Manzini Region	Shiselweni Region
Baylor Clinic	Cabrini Clinic	AHF Lamvelase Clinic	Gege Clinic
Bhalekane Clinic	Good Shepherd Hospital	Bulunga Clinic	Hlathikhulu Hospital
Dvokolwako Health Center	Kudvumisa Clinic	Cana Clinic	Hluthi Clinic
Mangweni Clinic	Lomahasha Clinic	Luyengo Clinic	JCI Clinic
Mbabane Government Hospital	Lubuli Clinic	Mafutseni Clinic	Kamfishane Clinic
Mkhuzweni Health Center	Siphofaneni Clinic	Mankayane Hospital	Kaphunga Clinic
Motshane Clinic	Siteki Nazarene Clinic	Ngculwini Clinic	Mashobeni Clinic
Ndvwabangeni Clinic	Sithobela Health Center	Raleigh Fitkin Memorial Hospital	Matsanjeni Clinic
Ngowane Clinic			New Haven Clinic
Nkhaba Clinic			Nhlangano Health Center
Ntfonjeni Clinic			Silele Clinic
Piggs Peak Hospital			

Study Populations

Criteria for inclusion of subjects

a) Adult clients (Survey) – Adults, age 18 and older, enrolled in care and DSD at the study sites who present for care during the implementation time frame will be asked to participate in the survey
b) Healthcare workers (Survey) – HCW's working in ART facilities offering DSD will be offered the HCW satisfaction survey
c) Healthcare workers (In Depth Interview) - HCWs most experienced with DSD in their facility will be purposively sampled based on recommendations by the Senior HCW in each facility
d) Healthcare workers (Site Survey) – A Senior HCW of each study site, will be selected to complete the structured site survey.

Criteria for exclusion of subjects

a) Adult clients (Survey)- HIV negative, HIV positive adults not enrolled in a DSD model or younger than 18 years of age
b) Healthcare workers (Survey) – HCW's working in facilities not offering DSD; HCW working in DSD facilities who do not provide ART services
c) Healthcare workers (In Depth Interview) - HCW's working in facilities not offering DSD; HCW working in DSD facilities who do not provide ART services
d) Healthcare workers (Site Survey) – HCW's working in facilities not offering DSD, junior HCWs in a facility offering DSD, HCWs without a broader picture of the facility

Sample Size

For the client and HCW satisfaction surveys, a minimum of 6 clients and 3 HCWs will be surveyed per study site. Clients will be selected using a purposive sampling approach, with the aim to enroll at least one client from each of the 6 possible DSD models available within the respective site. The overall sample sizes will include at least 234 clients and 117 HCWs from 39 health facilities. One senior HCW will be requested to participate in a Senior HCW Survey for their facility (N=39). In addition, at least 15 HCWs in total, selected from the 39 health facilities, will participate in HCW In-depth Interviews.

Recruitment and enrollment

Client Satisfaction Survey

For the Client Satisfaction Survey, clients 18 years of age and older who are enrolled in any of the DSD models will be eligible for the survey when presenting for care and treatment during the data collection period and will be offered the opportunity to complete the survey at study sites on days that study staff members are present. Some aspects of purposive selection, recruitment, and enrollment procedures will differ by client DSD model, as described below. Across models, participants will be invited to participate by a HCW at the conclusion of a clinical visit in a facility or outreach location, or, for Community ART Group clients, by telephone. Using a standard recruitment script (Appendix 12), HCWs will inform DSD clients of the survey and invite them to speak to the data collectors if they are interested in participating. Prospective participants will be led to a private space for eligibility assessment and informed consent. During the informed consent process data collectors will read aloud the informed consent script (Appendix 6), including a detailed description of the purpose, objectives, and procedures in siSwati or English and answer any questions prospective participants have. If prospective participants affirm that they are fully informed about the study and survey and wish to participate, study staff will confirm their wish according to the information sheet, and, if agreeing to participate, verbal consent will be provided by the client. In order to protect the anonymity of participants, names or other identifying information will not be collected at any point in the informed consent process or during the survey. No compensation will be given to clients who participate.

Recruitment procedures will be tailored by each DSD model in the following ways:

- **ART Fast Track, Facility Treatment clubs and Family Centered Care Model**: During the three-month data collection period, study staff will instruct facility HCWs to identify eligible (i.e., age 18 years or older) clients enrolled in ART Fast Track, Facility Treatment clubs or Family Centered Care Models, and introduce the satisfaction survey to the client. at the end of their routine clinical visit using the standard recruitment script as described above. If the client chooses to participate, study staff will perform consent procedures and administer the survey.
- **Community ART Groups:** CAG members will be identified by HCWs by reviewing standard MoH tools including the DSD register and the Chronic Care File and contacted by telephone by a HCW and read the standard recruitment script over the phone. If the CAG member is interested in participating, the HCW will request permission for the client to be contacted by the study staff to schedule an appointment with the client at the next CAG meeting. The study staff will meet the CAG member during the scheduled group meeting facilitated by a mentor or expert client. The study staff will introduce the study to the group and clients that previously agreed to be surveyed will be requested to remain behind after the meeting. Participating CAG members will then be escorted to a private area to conduct consent procedures and have the survey administered by study staff.
- **Outreach:** Study staff will accompany mobile clinical teams from the health facility to outreach sites. Mobile HCWs will introduce the survey to clients enrolled in the outreach model at the end of their routine clinical visit using the standard recruitment script as described above. If the client chooses to participate, study staff will perform consent procedures and administer the survey.

Following verbal informed consent, study staff will read survey questions aloud to participants and record their answers on an electronic tablet. Survey participants will be recruited over an eight to twelve-week period until the sample size is reached within each facility.

Healthcare Worker Satisfaction Survey

For the HCW Satisfaction Survey, all HCWs working in ART clinics within facilities offering DSD during the implementation period will be offered the opportunity to complete the survey at study sites. Study staff will set a facility appointment through the Senior HCW to gather all facility staff in one room of the facility at a time of their convenience.

The study staff will read a recruitment script (Appendix 12) and ask interested HCWs to remain in the room to perform consent procedures using the consent script in English (Appendix 8) and answer any questions prior to obtaining verbal informed consent. The data collector will then administer the paper-based HCW satisfaction surveys to the group simultaneously. This group recruitment and enrolment method is preferred to minimize impact on client service delivery at study sites as client queues are typically finished by lunch time and the HCWs can be gathered easily.

If it is not feasible to conduct a group survey administration for HCWs at a study site, data collectors will approach HCWs individually to schedule a survey time at the convenience of the HCW on the day of data collection. At the agreed upon time, the data collector will read a recruitment script (Appendix 12) to determine interest and escort interested HCW to a private space for informed consent and data collection. In a private space, the data collector will read aloud the consent script in English (Appendix 8) to eligible HCWs and answer any questions prior to obtaining verbal informed consent. Following informed consent, the data collector will give paper surveys to HCWs to complete . No compensation will be given to HCWs who participate. HCW names will not be linked to participation or responses in any records.

Healthcare Worker In-Depth Interviews

For the in-depth interviews, HCWs identified by the Senior HCW at study sites will be approached by the study staff and asked to participate in an in-depth interview at a time of their convenience. HCW will be met by a study staff at an agreed-upon time in a private area after client queues have been addressed to minimize potential impact on client care. Study staff will administer written consent procedures by reading aloud the consent script in English to eligible HCWs and answer any questions prior to signing two copies of the consent form (Appendix 10 & 11). One copy will remain with the HCW and the second copy will be kept by the study staff for study records.

For HCWs that provide written consent, the study staff will conduct the in-depth interview. The in-depth interview will be audio recorded to allow for later translation to English, if conducted in siSwati, and transcribed for analysis. No compensation will be given to HCWs who participate. HCW names will not be linked to participation or responses in any records.

Senior Healthcare Worker Site Survey

The Senior HCW at each study site will be approached by the study staff. Study staff will sit with the Senior HCW and read aloud the verbal consent script in English. Should the HCW choose to participate, they will be requested to complete a paper-base site survey at a time of their convenience No compensation will be given to the senior HCW who choses to participate. HCW names will not be linked to participation or responses in any records.

Procedures

Client Satisfaction Survey Administration

Clients age 18 years and older will complete verbal consent procedures with the study staff. This information will be provided to the client in the form of a participant information sheet to be read by the client or with assistance from study staff, depending on client preference (Appendix 6 and 7). After reviewing the participant information sheet, clients will provide verbal consent to the study staff prior to completion of the survey. The client survey will take approximately 25 minutes and will be administered by the study staff using a tablet. Participant responses will be recorded on the tablet by the study staff member during the interview.

Clients 18 years of age and older who are enrolled in any of the DSD models will be eligible for the survey. The survey will be conducted in siSwati or English.

Healthcare Worker Satisfaction Survey Administration

Once the HCWs are gathered in one location in the facility, the study staff will conduct a group introduction to the study and consent procedures. HCWs that consent to participate will self-administer the survey via pen and paper simultaneously

at the scheduled time with their colleagues, or individually based on availability, to minimize disruption to routine facility activities. The survey will take approximately 25 minutes.

Healthcare worker In-Depth Interviews

In-depth interviews with HCWs will take place at a private location within the premises of the health facility. Written consent will be obtained by study staff prior to beginning the interview that will be conducted in English or siSwati depending on the preference of the HCW. Prior to beginning the interview, the study staff member will complete a paper-based interview cover sheet that will capture:

- A unique interview code comprised of the first three letters of the facility, the date of the interview, sequential interview number and initials of the interviewer
- Facility name
- Interview date
- HCW cadre
- Duration of experience the HCW has with DSD

The interview will last approximately 30 minutes and be audio recorded to allow for later translation to English, if conducted in siSwati, and transcribed for analysis. Following the interview, the study staff will document observations from the interview and duration of the audio recording on the cover sheet that will be transported back to the Clinton Health Access Initiative office for secure filing.

Senior Healthcare Worker Site Survey Adminsitration

The Senior HCW at a study site will be approached by the study staff and asked to participate in a self-administered paper-based site survey, at a time of their convenience in a private room. Study staff will read verbal consent procedures in English. The survey will take approximately 20 minutes.

Procedures for Adverse Events

If a client or HCW becomes distressed as a result of answering survey questions, the survey will be stopped and the study staff assisting with the survey will address the needs of the client or HCW. Study staff will console the client or HCW and, if needed, refer them to counseling or other supportive services. Study staff will be trained on where these services are available and how to effectively link the client or HCW to these services if necessary. Any adverse events will be reported to the relevant institutional review boards.

Data Management and Storage

Mobile Data Collection System

SurveyCTO will be the mobile data collection platform used to securely upload survey files from the tablets to the dedicated SurveyCTO study server at the end of each day. SurveyCTO is certified by Columbia University Medical Center (CUMC) Information Technology as a multi-user platform for storing data. SurveyCTO is hosted on Amazon cloud servers (https://aws.amazon.com/) and the study team will be assigned a dedicated study server from SurveyCTO to be managed within the SurveyCTO platform. Only study personnel will have access to the encrypted password-protected database on the server. SurveyCTO aggregates data from completed collection forms into encrypted .csv files that can then be decrypted on a secure computer, and imported into software for analysis. All study tablets will be stored in a locked cabinet at the Clinton Health Access Initiative office when not in use

Client Satisfaction Survey

The client surveys will be captured directly by study staff into a SurveyCTO mobile data collection form using password protected and encrypted tablets. Client Satisfaction Survey electronic responses will be securely uploaded to the SurveyCTO server at the end of each day of data collection and the data removed from the tablet.

Healthcare Worker Satisfaction Survey

The paper-based, self-adminsitered HCW surveys will be transported from health facilities to the Eswatini Clinton Health Access Initiative office in a secure fashion by study staff, where they will be electronically captured by study staff via tablets using the SurveyCTO application. All paper HCW surveys will be stored in a locked cabinet at the Clinton Health Access Initiative office when not in use. In addition, all tablets will be password-protected and encrypted. All survey data will be electronically uploaded to a secure password protected SurveyCTO server on the day of survey completion and the data will be removed from the tablets.

Healthcare worker In-Depth Interviews

In-depth interview audio files will be transferred from the recording device to one password-protected and encrypted computer accessible only to study staff. Audio files will be deleted from the recording device after the files have been transferred to the computer.

The audio files will be transferred to the encrypted computer of the study translator/transcriber who will transcribe the interview into Word documents. Interview audio files and transcription files will be linked by a de-identified interview ID that includes a three-letter facility code, date of interview and initials of the interviewer. The transcript files will be stored on the same computer indicated above after transcription is completed and the audio files will be deleted by the study coordinator after transcription and a quality check have been concluded.

Senior Healthcare Worker Site Survey Adminsitration

The paper-based, self-adminsitered Senior HCW survey will be transported from health facilities to the Eswatini Clinton Health Access Initiative office by study staff, where they will be electronically captured by study staff into tablets using the SurveyCTO application. All paper Senior HCW surveys will be stored in a locked cabinet at the Clinton Health Access Initiative office when not in use. All survey data will be electronically uploaded to the secure password protected SurveyCTO server on the day of survey completion and the data will be removed from the tablets.

Data Sharing

All study data will be held by the the study coordinator with access by the Prinicipal Investigator. The data will be kept confidential by those involved directly in coordinating and conducting the study. Data access requests will be authorized by the Principal Investigator.

Study data collection progress tracker

To monitor progress towards client and HCW survey, and in-depth interview, enrollment targets, a password-protected Excel-based tracker will be implemented. No client or HCW worker names or other identifying information will be entered on the tracker. Number of clients enrolled by DSD model and number of HCWs by cadre, by facility will be updated daily on the tracker for both surveys and in-depth interviews to monitor data collection progress. This tracker will be maintained by and accessible only to the study coordinator on her/his encrypted laptop and deleted at the end of the data collection period.

Storage of Paper-Based Tools

Paper-based surveys, written consent forms and in-depth interview cover sheets will be stored in a locked filing cabinet at the Clinton Health Access Initiative office only accessible by study staff when not in use. At the end of the study, all paper-based study documents will be transferred to the Eswatini Ministry of Health for long-term storage in-line with institutional review board requirements.

Data backup

All electronic study data including the SurveyCTO exported client and HCW satisfaction survey results, in-depth interview audio recordings awaiting transcription and transcripts will be backed up once per week to an encrypted external hard

drive by the study coordinator. The external hard drive will be stored in a locked filing cabinet in the Clinton Healht Access Initiative office when not in use.

Data Analysis

Analysis of survey data will include descriptive statistics such as frequency of responses for each item and include comparisons across subgroups, including but not limited to: age group, sex, educational attainment, facility type, type of DSD model, type of ART regimen. Excel and Stata will be used to analyze quantitative data.

The qualitative data arising from the IDIs will be analysed through standard thematic analyses, identifying repeated ideas, themes, and thematic narratives through coding and synthesis at increasingly higher levels of abstraction. Dedoose or ATLAS.ti will be used as the platform for qualitative data analysis.

Timeline

Data collection for this study will take place across a 3-month study implementation period, followed-by data analysis and dissemination.

	2019									
	Month 1	Month 2	Month 3	Month 4	Month 5	Month 6	Month 7	Month 8	Month 9	Month 10
Protocol Development	X	X	X							
IRB Approval				X	X					
Study Preparation				X	X					
Study implementation					X	X	X			
Data cleaning							X	X		
Data analysis								X		
Report Preparation									X	
Data Dissemination										X

Ethical Considerations

Human Subjects

The study protocol will be submitted for review by the Eswatini National Health Research Review Board (NHRRB) and the Columbia University Irving Medical Center Institutional Review Board.

Participation in the survey is voluntary; all participants approached for participation will be informed of the voluntary nature. Potential participants will also be informed that they will receive care at the facility regardless of their decision to complete the survey. HCWs will be informed that participation in the study through surveys or in-depth interviews will not impact their employment in any way.

Informed Consent

Informed consent for surveys

For the surveys, Clients, HCWs and Senior HCWs who are 18 years of age and older will be eligible for informed verbal consent. When the client or HCW is contacted by study staff, they will first read the appropriate study script in appendix 12. Upon agreement, the study staff will read aloud in English or siSwati the consent prcedures which include a description

of the purpose, objectives and procedures related to the survey. Study staff will review the content of the informed verbal consent information sheet with all participants to ensure an adequate understanding of the survey. Participants that are literate in siSwati or English will also be given the information sheet to read and the chance to ask any questions. Participants will be informed that they do not have to participate in the survey at all if they choose not to and, if they choose to participate, they may also voluntarily withdraw from the study for any reason and at any time. The participant will be offered a hard copy of the verbal consent information sheet to keep. The verbal consent information sheets are included as appendices 6-9.

The research activities do not involve any interaction or interference with routine care and participation in the survey will not alter in any way the routine services that all clients receive. The survey is anonymous and no client identifiers will be collected nor will survey data be linked to medical records.

Informed consent for in-depth interviews

For in-depth interviews, HCWs 18 years of age and older will be approached by study staff. Study stuff will read aloud in English or Siswati the consent prcedures which include a description of the purpose, objectives and procedures related to the survey. Study staff will review the content of the informed written consent information sheet with all participants to ensure an adequate understanding of the survey. Participants that are literate in siSwati or English will also be given the information sheet to read and the chance to ask any questions. Participants will be informed that they do not have to participate in the survey at all if they choose not to and, if they choose to participate, they may also voluntarily withdraw from the study for any reason and at any time. The participant will sign the written consent form and can retain copy of the written consent information sheet. The written consent information sheets are included as appendices 10-11.

Anonymous Participation

Clients, HCWs (surveys and in depth interviewees) and Senior HCWs participating in the site surveys' names will not be documented on the surveys or tablets. All surveys and in-depth Interview cover sheets will be assigned a unique, anonymized survey number by the study team that will not contain any participant identifiers. This survey number will be used to uniquely identify the surveys within the electronic study database.

Request for waiver of written consent

A waiver of written consent will be requested from the institutional review boards for the client surveys, HCW surveys and senior HCWs' site surveys. The rationale for waiving written consent for these groups is that the study presents no more than minimal risk to these participants and the survey data will be anonymous. Therefore, the written consent form would be the only record linking the participants and the study and the principal risk would be potential harm resulting from a breach of confidentiality. As indicated previously, these participants will receive an information sheet containing the appropriate elements of consent disclosure and the study staff will obtain and indicate verbal consent prior to survey administration.

Risks and Benefits of Participation

The study poses no more than minimal risk to the participants because the only known risk to clients and HCWs is the possible loss of confidentiality which will be protected against by collecting minimal personally identifiable information including age, sex and DSD model and ensuring safety of data through encryption and password protection of electronic study data and limiting access to approved study staff as described in the 'Data Management and Storage' section above.

There are no direct benefits to clients whose data is included in the study.

Data Ownership

All data collected through this study will be owned by the Eswatini Ministry of Health National AIDS Program. Permission to use the data for secondary data analysis will be granted by the Ministry of Health and Eswatini NHRRB.

Expected Application of the Results

A final report of this study will be submitted to MOH, RHMTs and to implementers. The results of the study will also have relevance primarily for clinical providers who care for HIV-infected adults and adolescents and policymakers who oversee program implementation and develop care and treatment guidelines.

Efforts to disseminate the findings of the study outside of Eswatini will include the publication of papers in scientific journals and presentation of findings at public health-related conferences and meetings. The investigators involved with the study will also participate in and contribute data for guideline development processes to assist in the full effective utilization of the findings. Information on the findings relevant for wider audiences, including the general public, will also be disseminated through press releases to print and other media outlets if deemed relevant by the MOH. All study publications must be approved by the Principal Investigator prior to circulation.
